# Trends in the kidney cancer mortality-to-incidence ratios according to health care expenditures of 56 countries

**DOI:** 10.1038/s41598-020-79367-y

**Published:** 2021-01-14

**Authors:** Wen-Wei Sung, Po-Yun Ko, Wen-Jung Chen, Shao-Chuan Wang, Sung-Lang Chen

**Affiliations:** 1 Department of Urology, Chung Shan Medical University Hospital, Taichung, 40201 Taiwan; 2 School of Medicine, Chung Shan Medical University, Taichung, 40201 Taiwan; 3 Institute of Medicine, Chung Shan Medical University, Taichung, 40201 Taiwan

**Keywords:** Health care, Urology

## Abstract

The incidence and mortality rates in kidney cancer (KC) are increasing. However, the trends for mortality have varied among regions over the past decade, which may be due to the disparities in medical settings, such as the availability of frequent imaging examinations and effective systemic therapies. The availability of these two medical options has been proven to be positively correlated with a favorable prognosis in KC and may be more common in countries with better health care systems and greater expenditures. The delicate association between the trends in clinical outcomes in KC and health care disparities warrant detailed observation. We applied a delta-mortality-to-incidence ratio (δMIR) for KC to compare two years as an index for the improvement in clinical outcomes and the mortality-to-incidence ratio (MIR) of a single year to evaluate their association with the Human Development Index (HDI), current health expenditure (CHE) per capita, and CHE as a percentage of gross domestic product (CHE/GDP) by using linear regression analyses. A total of 56 countries were included based on data quality reports and missing data. We discovered that the HDI, CHE per capita, and CHE/GDP were negatively correlated with the MIRs for KC (*p* < 0.001, *p* < 0.001, and *p* < 0.001, respectively). No significant association was observed between the δMIRs and the HDI, CHE per capita, and CHE/GDP among the included countries, and only the CHE/GDP shows a trend toward significance. Interestingly, the δMIRs related with an increase in relative health care investment include δCHE per capita and δCHE/GDP.

## Introduction

In 2018, kidney cancer (KC) was the 15th most common newly diagnosed cancer; it claimed the lives of 403,262 individuals and accounted for 2.2% of all cancers. Furthermore, KC had the 17th highest cancer-related mortality with 175,098 deaths, which accounts for 1.8% of all cancer-related deaths worldwide^1^. By observing the statistics for KC since 2012^2^, a slight growing trend can be seen for both new cases and KC-related deaths. This finding suggests that the disease still warrants further investigation for better prevention and treatment. The geographic distribution of KC shows mainly higher incidence in Eastern European countries, such as Slovakia and the Czech Republic, while lower incidence is seen in Africa and Asia, with the exception of Israel. The distribution of KC-related mortality generally follows the incidence pattern, with the highest death rates in Eastern Europe^3^.

Clinically, the diagnosis of KC is usually incidental and mainly attributed to various imaging modalities, such as ultrasonography, computed tomography, and magnetic resonance imaging (MRI)^4^. Moreover, a small renal mass (SRM, < 4 cm in size) that is detected by imaging usually needs to be further examined to differentiate KC from benign lesions, such as a pure cyst, oncocytoma, or angiomyolipoma, since early and precise diagnosis can make a life-changing impact on the prognosis of individuals with KC^5^. More than 90% of KC cases are of the renal cell carcinoma (RCC) pathological type, with the main subtype being clear cell RCC^6^. The treatment options for RCC depend on the stage at detection. An early diagnosis is usually treated with partial nephrectomy and associated with favorable outcomes^7,8^. Conversely, an advanced stage of RCC and the metastasis of RCC at diagnosis are often linked to a poor prognosis and recurrent disease, which warrants systemic treatment rather than mere local excision^7,9,10^. Over the past decade, treatments for the management of advanced clear cell RCC, which is the most common subtype of KC, have progressed tremendously. Several immunotherapy agents that use tyrosine kinase inhibitors (TKIs), such as sunitinib and pazopanib, have been demonstrated to provide a dramatic improvement in progression-free survival^11,12^. This revolutionary progress in treatments may contribute to a better overall survival rate for KC.

The clinical outcome of diseases is usually measured not only by 5-year-survival rate but also by the mortality-to-incidence ratio (MIR)^13–17^. Over the past decade, the incidence rates of RCC have been increasing in most countries, with the greatest rise in Latin America^18^. Although the total number of deaths from KC has increased, the heterogeneity of the mortality is notable among regions. Several countries in Western and Northern Europe have shown a stable mortality trend, while countries such as the United States and Australia have demonstrated significant declines in mortality^18^. Thus, the MIR may have a role in further evaluating improvements in RCC outcomes. Moreover, this variation among regions also implies that health care systems and health care expenditures make an impact on the early diagnosis, effective treatment, and prognosis for KC.

In this study, we set our primary goal to become the first study to evaluate dynamic trends in MIR by observing the changes between the MIR values for GLOBOCAN 2012 and GLOBOCAN 2018 by subtracting the figure for 2018 from that for 2012. We then sought to determine if the delta-mortality-to-incidence-ratio (δMIR) had a correlation with the Human Development Index (HDI), current health expenditure (CHE) per capita, and CHE as a percentage of gross domestic product (CHE/GDP). The secondary goal was to identify the association between the MIR for KC and the expenditures of health care systems among the selected countries by utilizing the data from GLOBOCAN 2018. The expenditures of the health care systems were divided by HDI, CHE per capita, and CHE/GDP. We hypothesized that both the MIR and the δMIR would positively correlate with higher values for HDI, CHE per capita, and CHE/GDP.

## Materials and methods

### Epidemiological materials

The epidemiological data for KC were obtained from the GLOBOCAN project (http://gco.iarc.fr/), which is maintained by the International Agency for Research on Cancer of the World Health Organization (WHO). This database provides epidemiological information for 185 countries. The data for health expenditures, including CHE per capita and CHE/GDP, were obtained from the World Health Statistics (https://www.who.int/gho/publications/world_health_statistics/en/) of the WHO (obtained on May 16, 2020). The HDI was obtained from the United Nations Development Programme’s Human Development Report Office (http://hdr.undp.org/en) (obtained on May 16, 2020). In this analysis, the exclusion criteria for country selection was based on the data quality report from GLOBOCAN (N = 121), missing data (N = 3), and outliers for MIR/δMIR (N = 5). A total of 56 countries were included in the final analysis. The MIR was defined as the ratio of the crude rate (CR) of mortality and the CR of incidence, as described previously^19–22^. The δMIR was defined as the difference between the MIR of 2012 and the MIR of 2018 (δMIR = MIR [2012]—MIR [2018])^23^. δHDI, δCHE per capita and δCHE/GDP were defined as the difference between the values of 2012 and the values of 2018. The age standardized rates (ASR) were analyzed with ages that ranged from 0 to 84 years old.

### Statistical analyses

The associations among the incidence, mortality, MIRs, δMIRs, and other factors of the various countries were estimated by a Spearman's rank correlation coefficient using SPSS statistical software, version 15.0 (SPSS, Inc., Chicago, IL). *P* values of < 0.05 were considered statistically significant. A scatterplot was generated using Microsoft Excel.

### Consent for publication

The authors agree the publication after official review and acceptance.

## Results

### Epidemiology of kidney cancer across regions

We intended to understand the present global situation of KC by employing data from the GLOBOCAN 2018 database (Table 1). The distribution of KC according to continent was obtained and analyzed. There were 403,262 new cases of KC and 175,098 deaths from KC worldwide, which accounts for 2.5% among all types of cancer. Among the different regions, Asia had the largest numbers for both incidence and mortality (148,947 and 79,149, respectively), while Oceania had the lowest incidence and mortality (5018 and 1564, respectively).

**Table 1:** Summary of the KC incidence, mortality, and MIR according to regions.

Region	Incidence			Mortality			MIR
	Number	CR	ASR	Number	CR	ASR	
**HDI**							
Very High HDI	239,567	17.3	9.4	82,786	6.0	2.6	0.35
High HDI	49,127	4.9	3.6	22,118	2.7	1.9	0.55
Medium HDI	19,677	1.3	1.4	11,270	0.77	0.88	0.59
Low HDI	8843	0.86	1.1	5467	0.53	0.85	0.62
**Continent**							
Africa	13,420	1.0	1.4	8130	0.63	0.96	0.63
Asia	148,947	3.3	2.8	79,149	1.7	1.4	0.52
Europe	136,515	18.4	9.6	54,709	7.4	3.1	0.40
Latin America and the Caribbean	31,938	4.9	4.4	14,288	2.2	1.9	0.45
North America	67,424	18.5	10.9	17,258	4.7	2.3	0.25
Oceania	5018	12.2	8.3	1564	3.8	2.2	0.31

In terms of the age standardized rate for incidence, North America was shown to have the highest ASR (10.9) followed by Europe, while Africa had the lowest ASR (1.4). However, the ASR for mortality indicated that Europe had the highest ASR (3.1) followed by North America (2.3). Africa had the lowest ASR (0.96). The lowest MIR in 2018 was seen in North America (0.25) followed by Oceania (0.31), and the highest MIR was documented in Africa (0.63).

### Epidemiology and disparities in health expenditures and development according to country

In this study, we aimed to consider the differences among the 56 countries by including countries based on their national data from the World Health Statistics from the WHO. The HDI, CHE per capita, CHE/GDP, CR of incidence and mortality, and ASR of incidence and mortality are listed in Table 2. The highest HDI was obtained in Norway (0.953), while the lowest HDI was obtained in Egypt (0.696). The CHE/GDP ranged from 3.1% in Qatar to 16.8% in the United States.

**Table 2:** Summary of HDI, CHE, cancer incidence, cancer mortality, and MIR in KC of selected countries (N = 56).

Country	HDI		CHE		Incidence			Mortality			MIR		
	Score	Rank	Per capita	% of GDP	ASR	CR	Cum. risk	ASR	CR	Cum. risk	2012	2018	δMIR
Argentina	0.825	47	998	6.8	8.5	10.5	1.04	3.6	4.8	0.45	0.49	0.46	0.03
Australia	0.939	3	4934	9.4	9.5	15.6	1.13	2.1	4.0	0.25	0.27	0.26	0.01
Austria	0.908	20	4536	10.3	6.9	14.1	0.86	2.2	5.7	0.28	0.41	0.40	0.01
Belarus	0.808	53	352	6.1	16.7	27.1	1.97	6.4	11.2	0.80	0.41	0.41	0.00
Belgium	0.916	17	4228	10.5	9.1	17.0	1.11	2.2	5.1	0.27	0.41	0.30	0.11
Brazil	0.759	79	780	8.9	4.2	4.9	0.48	1.5	1.8	0.17	0.53	0.37	0.16
Bulgaria	0.813	51	572	8.2	6.4	11.8	0.74	2.5	5.4	0.31	0.54	0.46	0.08
Canada	0.926	12	4508	10.4	10.0	18.0	1.19	2.0	4.3	0.25	0.31	0.24	0.07
Chile	0.843	44	1102	8.1	7.1	10.0	0.88	3.1	4.8	0.39	0.54	0.48	0.06
Colombia	0.747	90	374	6.2	3.2	3.5	0.37	1.1	1.3	0.14	0.45	0.37	0.08
Costa Rica	0.794	63	929	8.1	3.3	4.1	0.39	1.3	1.7	0.15	0.38	0.41	− 0.03
Croatia	0.831	46	852	7.4	11.5	22.0	1.36	3.7	8.9	0.45	0.46	0.40	0.06
Cuba	0.777	73	826	10.9	4.3	7.1	0.51	1.4	2.5	0.17	0.52	0.35	0.17
Cyprus	0.869	32	1563	6.8	4.8	7.3	0.57	1.8	3.2	0.20	0.37	0.44	− 0.07
Czechia	0.888	27	1284	7.3	14.3	29.1	1.80	4.3	9.9	0.54	0.33	0.34	− 0.01
Denmark	0.929	11	5497	10.3	9.1	17.4	1.10	2.5	5.9	0.31	0.47	0.34	0.13
Ecuador	0.752	86	530	8.5	3.2	3.2	0.37	1.2	1.2	0.14	0.56	0.38	0.18
Egypt	0.696	115	157	4.2	2.2	1.9	0.25	1.3	1.1	0.15	0.71	0.58	0.13
Estonia	0.871	30	1112	6.5	14.3	29.3	1.82	4.2	10.4	0.52	0.50	0.35	0.15
Finland	0.920	15	4005	9.4	7.9	16.7	0.95	2.4	6.3	0.30	0.38	0.38	0.00
France	0.901	24	4026	11.1	12.2	22.6	1.46	2.8	6.3	0.33	0.38	0.28	0.10
Germany	0.936	5	4592	11.2	8.5	18.6	1.02	2.9	8.4	0.35	0.41	0.45	− 0.04
Iceland	0.935	6	4375	8.6	11.9	19.3	1.49	2.7	5.1	0.32	0.42	0.26	0.16
Ireland	0.938	4	4757	7.8	10.9	17.4	1.31	2.4	4.4	0.30	0.40	0.25	0.15
Italy	0.880	28	2700	9.0	8.8	18.9	1.05	2.0	5.4	0.24	0.37	0.29	0.08
Jamaica	0.732	97	294	5.9	1.4	1.6	0.17	0.75	0.87	0.09	0.64	0.54	0.10
Japan	0.909	19	3733	10.9	6.9	16.9	0.83	1.4	4.7	0.17	0.48	0.28	0.20
Kuwait	0.803	56	1169	4.0	2.3	1.6	0.30	1.1	0.62	0.15	0.42	0.39	0.03
Latvia	0.847	41	784	5.8	14.8	29.1	1.79	4.2	10.6	0.52	0.50	0.36	0.14
Lithuania	0.858	35	923	6.5	14.5	27.8	1.76	4.8	10.8	0.60	0.40	0.39	0.01
Luxembourg	0.904	21	6236	6.0	6.6	10.4	0.82	1.2	2.4	0.14	0.17	0.23	− 0.06
Malaysia	0.802	57	386	4.0	2.7	2.7	0.32	1.4	1.4	0.15	0.43	0.52	− 0.09
Malta	0.878	29	2304	9.6	9.2	16.7	1.04	2.5	5.9	0.31	0.47	0.35	0.12
Mauritius	0.790	65	506	5.5	2.4	3.3	0.27	0.86	1.3	0.11	0.48	0.39	0.09
Netherlands	0.931	10	4746	10.7	7.5	14.8	0.92	2.9	6.6	0.36	0.55	0.45	0.10
New Zealand	0.917	16	3554	9.3	8.3	13.8	1.00	2.4	4.7	0.30	0.34	0.34	0.00
Norway	0.953	1	7464	10.0	9.9	17.4	1.22	2.1	4.4	0.26	0.33	0.25	0.08
Philippines	0.699	113	127	4.4	2.4	2.0	0.29	1.3	1.0	0.14	0.60	0.50	0.10
Poland	0.865	33	797	6.3	8.6	15.8	1.05	3.5	7.3	0.44	0.52	0.46	0.06
Portugal	0.847	41	1722	9.0	6.2	11.9	0.70	1.5	4.0	0.18	0.36	0.34	0.02
Qatar	0.856	37	2030	3.1	2.2	1.1	0.25	1.7	0.63	0.19	0.47	0.57	− 0.10
Russian Federation	0.816	49	524	5.6	9.9	16.2	1.19	3.5	6.4	0.45	0.47	0.40	0.07
Serbia	0.787	67	491	9.4	7.8	13.4	0.94	2.8	5.7	0.36	0.46	0.43	0.03
Singapore	0.932	9	2280	4.3	9.1	15.9	1.11	2.5	4.8	0.31	0.43	0.30	0.13
Slovakia	0.855	38	1108	6.9	13.1	22.8	1.60	4.4	8.8	0.55	0.37	0.39	− 0.02
Slovenia	0.896	25	1772	8.5	10.2	20.5	1.26	3.3	8.0	0.40	0.43	0.39	0.04
South Korea	0.903	22	2013	7.4	7.8	13.3	0.91	1.2	2.4	0.14	0.22	0.18	0.04
Spain	0.891	26	2354	9.2	8.5	16.3	1.01	2.2	5.0	0.26	0.36	0.31	0.05
Sweden	0.933	7	5600	11.0	6.7	12.5	0.82	2.0	5.1	0.25	0.57	0.41	0.16
Switzerland	0.944	2	9818	12.1	5.8	11.8	0.71	1.6	4.0	0.18	0.47	0.34	0.13
Thailand	0.755	83	217	3.8	1.3	2.0	0.16	0.76	1.1	0.09	0.60	0.55	0.05
Trinidad and Tobago	0.784	69	1146	6.0	2.3	3.2	0.31	1.3	1.8	0.20	0.54	0.56	− 0.02
Ukraine	0.751	88	125	6.1	8.9	15.1	1.07	3.4	6.4	0.44	0.49	0.42	0.07
United Kingdom	0.922	14	4356	9.9	9.8	18.5	1.17	2.3	5.3	0.28	0.43	0.29	0.14
United States of America	0.924	13	9536	16.8	10.7	17.6	1.27	2.1	4.0	0.26	0.26	0.23	0.03
Uruguay	0.804	55	1281	9.2	11.2	16.6	1.36	4.1	7.0	0.52	0.53	0.42	0.11

### Differences in incidence and mortality for kidney cancer according to country

We also identified the differences in the detailed information on KC. Belarus had the highest rates for ASR and the cumulative risk of both incidence and mortality for KC, and Thailand had the lowest ASR and cumulative risk of incidence, while Jamaica had similar incidence rates with a slightly lower ASR for incidence and the same cumulative risk for mortality. Moreover, we investigated the MIR data from 2012 and 2018. In 2012, of all the countries included, the highest MIR for KC was obtained in Egypt (0.71), and the lowest MIR for KC was obtained in Luxembourg (0.17). In 2018, the highest MIR occurred in Qatar (0.58), while the lowest MIR was recorded in South Korea (0.18). Furthermore, our study analyzed δMIR by subtracting the MIR in 2018 from the MIR in 2012. A positive value for this index may indicate an improvement in outcomes for KC. The highest value for δMIR was seen in Japan (0.20), and the lowest value for δMIR was documented in Qatar (-0.10).

### The association between the HDI, CHE per capita, and CHE/GDP and the crude rates of incidence and mortality in KC

**Figure 1:**
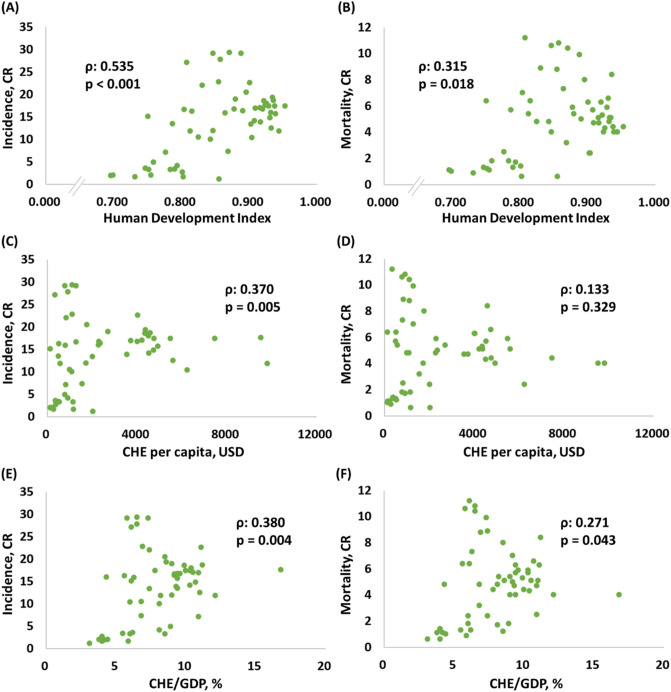
Association between HDI, CHE, and the crude rates of (**A**,**C**,**E**) incidence and (**B**,**D**,**F**) mortality in KC.

We further sought to investigate the association between HDI, CHE per capita, and CHE/GDP and the CRs of incidence and mortality of different countries for KC (Fig. 1). We discovered that crude incidence rates were significantly associated with higher HDI, CHE per capita, and CHE/GDP. Crude mortality rates had a significant correlation with a higher HDI and CHE/GDP but not CHE per capita, as shown in Fig. 1A–F.

### Significant correlations between MIRs and HDI, CHEs per capita, and CHEs in KC

**Figure 2:**
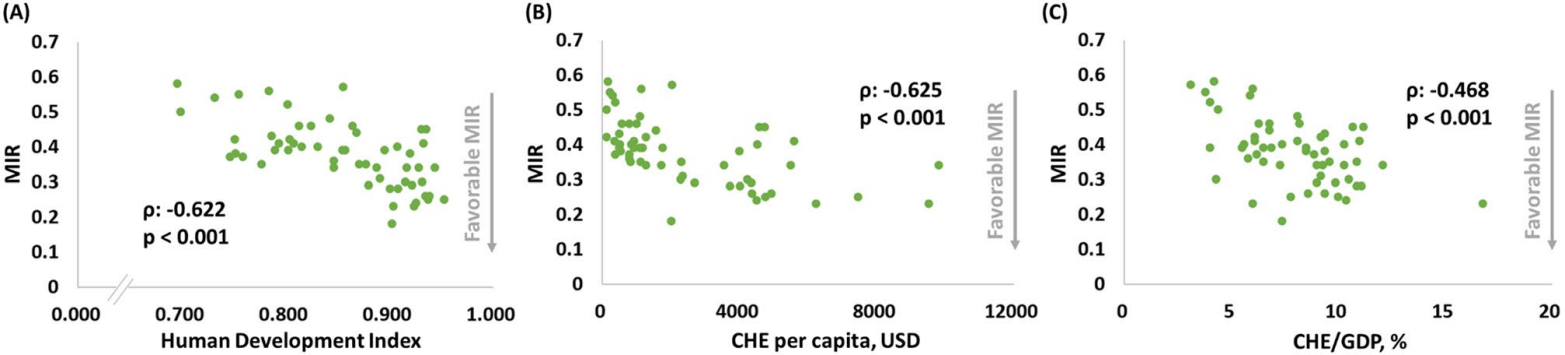
(**A**) HDI, (**B**) CHE per capita, and (**C**) CHE/GDP are significantly associated with the MIR in KC.

The significant associations between HDI, CHE per capita, and CHE/GDP and the MIRs for KC were also demonstrated. These indexes showed negative correlations with the MIRs for KC, as shown in Fig. 2A–C (ρ: − 0.622, p < 0.001, Fig. 2A; ρ: − 0.625, p < 0.001, Fig. 2B; and ρ: -0.468, p < 0.001, Fig. 2C).

### Associations between δMIRs and HDI, CHEs per capita, and CHEs in KC

**Figure 3:**
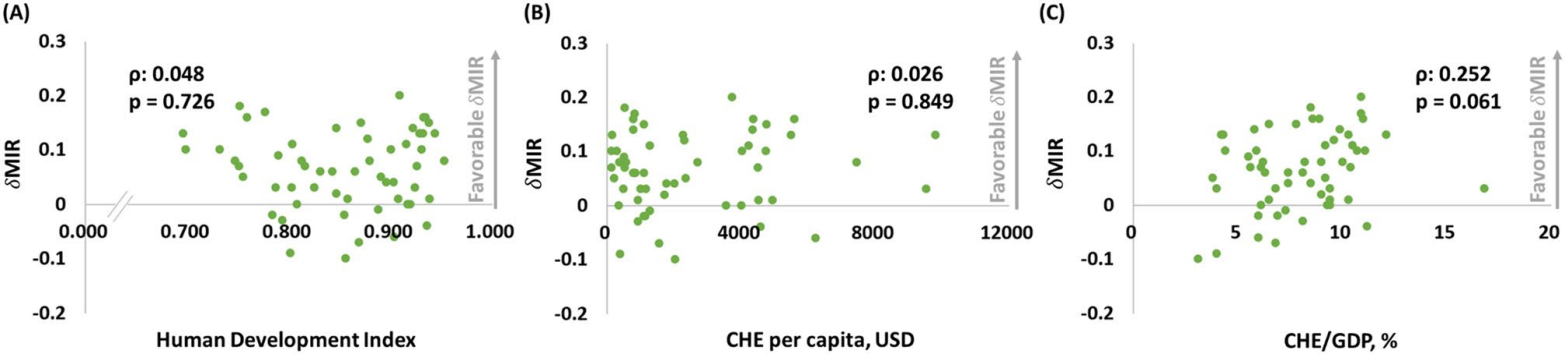
(**A**) HDI, (**B**) CHE per capita and, (**C**) CHE/GDP are not statistically associated with the δMIR in KC.

In this study, we also utilized δMIR to represent improved outcomes in KC and intended to elucidate the relationship between δMIR and the previously mentioned indexes (Fig. 3). However, HDI, CHE per capita, and CHE/GDP were not shown to have a significant correlation with δMIR, with only CHE/GDP that shows a trend toward statistical significance, as presented in Fig. 3A–C (ρ: 0.048, p = 0.726, Fig. 3A; ρ: 0.026, p = 0.849, Fig. 3B; and ρ: 0.252, p = 0.061, Fig. 3C). The improvement in outcome that is further related with an increase in relative health care investment showed that the δCHE per capita and δCHE/GDP were significantly correlated with δMIR, and only δHDI showed no statistical significance (δCHE per capita, n = 52, ρ: 0.303, p = 0.029; δCHE/GDP, n = 52, ρ: 0.322, p = 0.020; and δHDI, n = 56, ρ: 0.220, p = 0.104).

## Discussion

This study has been the first study to elucidate the relationship among health care system expenditures, HDI, and δMIR for KC worldwide, in addition to the more commonly explored association between health care expenditures and the MIRs of cancers^14,22,24,25^. We employed data from the GLOBOCAN database and World Health Statistics of the WHO. We aimed to identify the potential role of δMIR for predicting higher expenditures in a health care system as well as higher HDI among countries. The secondary goal was to reevaluate the known negative correlation between the MIR for KC and health care expenditures by utilizing the latest MIR data from 2018. In this study, we demonstrated a significant negative correlation between MIR and HDI, CHE per capita, and CHE/GDP that is concordant with previous findings using MIR data from 2012^21^. This correlation is also similar to the correlation obtained in several studies for other cancers, which have concluded that higher expenditures on health care systems are linked to lower MIRs^19,20,26^. Furthermore, the association between the δMIR of KC and HDI, CHE per capita, and CHE/GDP was examined, which has not been performed. We documented that the δMIR of KC was not significantly correlated with HDI, CHE per capita, or CHE/GDP, and only CHE/GDP has a trend toward statistical significance.

As with many malignancies, the prognosis for KC is prominently determined by the stage upon diagnosis^7,8^. Thus, early diagnoses have an important role in improving the mortality rate. The localized stage presents with a 93% 5-year survival rate, while the distant stage has a poor 5-year survival rate of just 12%^27^. However, unlike cancers with well-developed screening modalities, such as colorectal cancer or breast cancer^28,29^, KC is currently lacking a mature and cost-effective screening program, and most early diagnoses are due to unexpected detection by either ultrasonography or the cross-sectional imaging of an SRM, which is defined as an incidentally detected contrast-enhanced lesion of ≤ 4 cm^30^. This finding implies that countries with more frequent medical examinations and more regular access for individuals to undergo imaging procedures may have higher rates of early detection, and consequently, lower mortality rates. More intense medical programs require greater health expenditures, which would be reflected by indexes such as HDI, CHE per capita, and CHE/GDP.

Furthermore, the diversity of treatment options that have emerged over past decades cannot be overlooked when evaluating the improving trend for survival^31^. For many years, individuals with KC had been provided with few treatment options beyond surgery, and they rarely survived beyond 1 year^32^. However, this situation has changed over the past decade with an explosion of therapeutic treatments, which can mainly be divided into two different mechanisms that combat the malignancy^32^. The first set primarily controls the pathways for tumor growth and angiogenesis by interfering with genes and proteins, such as vascular endothelial growth factor receptor (VEGFR) inhibitors and mammalian target of rapamycin (mTOR) inhibitors, which are commonly employed for advanced RCC, which is the most common subtype of KC^33^. The second group includes immunotherapies, such as checkpoint inhibitors, that are able to enhance the mechanism of the body in fighting KC by activating T cells, which are known to have the function of not only killing a malignancy but also memorizing the cell and preventing new metastases from thriving again^34^. The constant development of systemic therapies has been proven to improve survival in KC in trials, especially in patients with an advanced stage^35,36^. In addition, the benefit is evidently remarkable even when some of them are utilized as second-line treatments after malignancy progression with initial therapies^37^.

The convincing explanation for the impact of high health care expenditures on satisfactory MIRs for KC is implied. A mature medical system that is able to provide more frequent imaging studies and easier access to efficient systemic therapeutic agents contributes to greater chances of early malignancy detection and effective curative treatments, which are two key elements for decreasing mortality. On the other hand, the incidence rate of KC has shown a universal trend of minimal change across countries over the years^1,3^. Hence, well-developed countries with greater expenditures on health care programs are reasonably connected to better MIRs for KC. Furthermore, this study, as a pioneer in utilizing the subtraction of the MIR in 2018 from the MIR in 2012, is capable of displaying more dynamic information about the improvement rate for clinical outcomes in KC. Taking advantage of the difference between these years, we were able to address the relationship between health care expenditures and the progress in combating KC. Although the results showed that only CHE/GDP had borderline statistical significance, which correlates with the δMIR, the importance of the δMIR can still not be overemphasized. A possible explanation includes that, despite the boom in systemic treatments such as VEGFR inhibitors and novel immunotherapy agents, vital issues such as proper patient selection and the optimal combination and sequencing of agents remain^38^. In addition, the ongoing large, multicenter, placebo‐controlled trials that explore the role of different adjuvant systemic agents in RCC indicate that treatment strategies still need improvement^38^. Therefore, the δMIR of KC is not significant in correlating with medical expenditures.

This study has some limitations. First, since countries with poor or unknown data quality were eliminated to prevent misleading effects, the results may not be legitimate enough to represent actual and precise global trends. Second, a few factors involved in the prognosis of KC were not included to obtain an accurate analysis, such as diagnosed stage, patient age, alcohol consumption, and obesity. Differences in medical settings, such as health care systems, insurance coverage, and individual habits of utilizing medical resources, should also be considered for a more thorough assessment. These conditions may cause differences in the incidence and mortality rates among countries. Furthermore, although we aimed to use δMIR to assess the constant change in clinical outcomes in KC, it still comprised a set of cross-sectional data that may not be valid for representing dynamic variations over years. Moreover, a previous study has disputed the validity of the MIR, which is being used as a surrogate for cancer survival, and points out the unstable difference between the MIR and cancer survival^39^. However, the study was conducted by using data on 20 cancers in England between 1981 and 2009. Conversely, our study exploits the latest data from 2018 in 56 countries, which may provide updated and extensive analyses compared to the prior study.

Data quality from this database is highly variable, and thus, this analysis was performed with global data with acceptable data quality. The results of the associations between HDI, CHE per capita, and CHE/GDP and the MIRs remained constant. The significant associations between HDI, CHE per capita, and CHE/GDP and the MIRs for KC were also demonstrated. These indexes showed negative correlations with the MIRs for KC, as shown in Fig. 2A–C (ρ: − 0.684, p < 0.001; ρ: − 0.698, p < 0.001; and ρ: − 0.305, p < 0.001, Fig. 2C). However, the associations between the factors and δMIR were changed. HDI, and CHE per capita were significantly correlated with δMIR, and only CHE/GDP show a trend toward statistical significance (ρ: − 0.512, p < 0.001; ρ: − 0.444, p < 0.001; and ρ: − 0.155, p = 0.047). Further analysis with other databases might clarify their association.

Despite the previously mentioned limitations, this study has succeeded in presenting a significant negative correlation between the HDI, CHE per capita, and CHE/GDP and the MIR, which was consistent with a previous study of the MIR for KC. Furthermore, the negative findings for an association between the δMIR and the HDI, CHE per capita, and CHE/GDP with CHE/GDP that presents a borderline significance were also established. Interestingly, the δMIRs related with an increase in relative health care investment include δCHE per capita and δCHE/GDP. Based on these results, the level of a health care system and expenditures may be useful for predicting the MIR for KC. The δMIR may still have a role in representing the progress in combating malignancies, and thus, deserves more attention for further illuminating its relationship with medical expenditures in the future.

## Conclusions

Favorable KC MIRs were significantly associated with high healthcare expenditures. Favorable KC δMIRs from 2012 to 2018 were associated with medical expenditures include δCHE per capita and δCHE/GDP.

## Data Availability

There was no additional unpublished data.

## References

[1] Bray, F. *et al.* Global cancer statistics 2018: GLOBOCAN estimates of incidence and mortality worldwide for 36 cancers in 185 countries. *CA Cancer J. Clin.* **68**, 394–424. 10.3322/caac.21492 (2018). 10.3322/caac.21492 30207593

[2] Ferlay J (2015). Cancer incidence and mortality worldwide: sources, methods and major patterns in GLOBOCAN 2012. Int. J. Cancer.

[3] Torre, L. A. *et al.* Global cancer statistics, 2012. *CA: A Cancer Journal for Clinicians* **65**, 87–108. 10.3322/caac.21262 (2015). 10.3322/caac.21262 25651787

[4] Rossi SH, Prezzi D, Kelly-Morland C, Goh V (2018). Imaging for the diagnosis and response assessment of renal tumours. World J. Urol..

[5] Heilbrun ME (2012). The cost-effectiveness of immediate treatment, percutaneous biopsy and active surveillance for the diagnosis of the small solid renal mass: Evidence from a Markov model. J. Urol..

[6] Rathmell WK, Godley PA, Rini BI (2005). Renal cell carcinoma. Curr. Opin. Oncol..

[7] Surveillance, Epidemiology, and End Results Program, National Cancer Institute. Cancer stat facts: Kidney and renal pelvis cancer. Available at https://seer.cancer.gov/statfacts/html/kidrp.html.

[8] Network NCC (2020). NCCN Guidelines: Kidney Cancer. Version.

[9] Cindolo L (2005). Comparison of predictive accuracy of four prognostic models for nonmetastatic renal cell carcinoma after nephrectomy: a multicenter European study. Cancer.

[10] Sorbellini M (2005). A postoperative prognostic nomogram predicting recurrence for patients with conventional clear cell renal cell carcinoma. J. Urol..

[11] Motzer RJ (2007). Sunitinib versus interferon alfa in metastatic renal-cell carcinoma. N. Engl. J. Med..

[12] Sternberg CN (2010). Pazopanib in locally advanced or metastatic renal cell carcinoma: results of a randomized phase III trial. J. Clin. Oncol..

[13] Sunkara V, Hebert JR (2016). The application of the mortality-to-incidence ratio for the evaluation of cancer care disparities globally. Cancer.

[14] Vrdoljak E (2019). Expenditures on oncology drugs and cancer mortality-to-incidence ratio in Central and Eastern Europe. Oncologist.

[15] Tsai MC (2017). Health disparities are associated with gastric cancer mortality-to-incidence ratios in 57 countries. World J. Gastroenterol..

[16] Lee HL (2018). Is mortality-to-incidence ratio associated with health disparity in pancreatic cancer? A cross-sectional database analysis of 57 countries. BMJ Open.

[17] Eberth JM (2019). Mortality-to-incidence ratios by US Congressional District: Implications for epidemiologic, dissemination and implementation research, and public health policy. Prev. Med..

[18] Znaor A, Lortet-Tieulent J, Laversanne M, Jemal A, Bray F (2015). International variations and trends in renal cell carcinoma incidence and mortality. Eur. Urol..

[19] Sunkara V, Hebert JR (2015). The colorectal cancer mortality-to-incidence ratio as an indicator of global cancer screening and care. Cancer.

[20] Chen SL (2017). Prostate cancer mortality-to-incidence ratios are associated with cancer care disparities in 35 countries. Sci. Rep..

[21] Sung WW (2018). Favorable mortality-to-incidence ratios of kidney Cancer are associated with advanced health care systems. BMC Cancer.

[22] Wang SC (2017). The gender difference and mortality-to-incidence ratio relate to health care disparities in bladder cancer: National estimates from 33 countries. Sci. Rep..

[23] Wang SC (2020). Limited improvement in prostate cancer mortality-to-incidence ratios in countries with high health care expenditures. Aging (Albany NY).

[24] Measuring performance on the Healthcare Access and Quality Index for 195 countries and territories and selected subnational locations: a systematic analysis from the Global Burden of Disease Study 2016. *Lancet* **391**, 2236–2271. 10.1016/s0140-6736(18)30994-2 (2018). 10.1016/S0140-6736(18)30994-2 PMC5986687 29893224

[25] Wang CC (2019). Favorable gallbladder cancer mortality-to-incidence ratios of countries with good ranking of world's health system and high expenditures on health. BMC Public Health.

[26] Wang CC (2017). Favorable liver cancer mortality-to-incidence ratios of countries with high health expenditure. Eur. J. Gastroenterol. Hepatol..

[27] Siegel, R. L., Miller, K. D. & Jemal, A. Cancer statistics, 2019. *CA Cancer J. Clin.* **69**, 7–34. 10.3322/caac.21551 (2019). 10.3322/caac.21551 30620402

[28] Greuter MJE, de Klerk CM, Meijer GA, Dekker E, Coupe VMH (2017). Screening for colorectal cancer with fecal immunochemical testing with and without postpolypectomy surveillance colonoscopy: A cost-effectiveness analysis. Ann. Intern. Med..

[29] Qaseem A, Lin JS, Mustafa RA, Horwitch CA, Wilt TJ (2019). Screening for breast cancer in average-risk women: a guidance statement from the American College of Physicians. Ann. Intern. Med..

[30] Sanchez A, Feldman AS, Hakimi AA (2018). Current management of small renal masses, including patient selection, renal tumor biopsy, active surveillance, and thermal ablation. J. Clin. Oncol..

[31] Capitanio U, Montorsi F (2016). Renal cancer. Lancet.

[32] Owens B (2016). Kidney cancer. Nature.

[33] Brown C (2016). Targeted therapy: An elusive cancer target. Nature.

[34] Schmidt C (2016). Immunotherapy: Controlled attack. Nature.

[35] Sanchez-Gastaldo A, Kempf E, Gonzalez Del Alba A, Duran I (2017). Systemic treatment of renal cell cancer: A comprehensive review. Cancer Treat Rev..

[36] Lee CH, Motzer RJ (2017). Kidney cancer in 2016: The evolution of anti-angiogenic therapy for kidney cancer. Nat. Rev. Nephrol..

[37] Tannir NM, Pal SK, Atkins MB (2018). Second-line treatment landscape for renal cell carcinoma: a comprehensive review. Oncologist.

[38] Barata PC, Rini BI (2017). Treatment of renal cell carcinoma: current status and future directions. CA Cancer J. Clin..

[39] Ellis L, Belot A, Rachet B, Coleman MP (2019). The mortality-to-incidence ratio is not a valid proxy for cancer survival. J. Global Oncol..

